# The Microbiota–Gut–Brain Axis in Metabolic Syndrome and Sleep Disorders: A Systematic Review

**DOI:** 10.3390/nu16030390

**Published:** 2024-01-29

**Authors:** Adriano dos Santos, Serena Galiè

**Affiliations:** 1 Integrative Medicine Nutrition Department, ADS Vitality B.V., 2517 AS The Hague, The Netherlands; 2 Department of Experimental Oncology, European Institute of Oncology IRCCS, 20139 Milano, Italy; serena.galie@ieo.it

**Keywords:** dysbiosis, gut microbiome, microbiome metabolites, metabolic syndrome, sleep quality, sleep efficiency

## Abstract

Background: Over recent decades, a growing body of evidence has emerged linking the composition of the gut microbiota to sleep regulation. Interestingly, the prevalence of sleep disorders is commonly related to cardiometabolic comorbidities such as diabetes, impaired lipid metabolism, and metabolic syndrome (MetS). In this complex scenario, the role of the gut–brain axis as the main communicating pathway between gut microbiota and sleep regulation pathways in the brain reveals some common host–microbial biomarkers in both sleep disturbances and MetS. As the biological mechanisms behind this complex interacting network of neuroendocrine, immune, and metabolic pathways are not fully understood yet, the present systematic review aims to describe common microbial features between these two unrelated chronic conditions. Results: This systematic review highlights a total of 36 articles associating the gut microbial signature with MetS or sleep disorders. Specific emphasis is given to studies evaluating the effect of dietary patterns, dietary supplementation, and probiotics on MetS or sleep disturbances. Conclusions: Dietary choices promote microbial composition and metabolites, causing both the amelioration and impairment of MetS and sleep homeostasis.

## 1. Introduction

In an ever-evolving landscape of scientific discovery, a growing body of evidence emerged in the last decade, delving into the symbiotic relationship between the gut microbiota and the delicate orchestration of sleeping patterns. 

Despite sleep being a physiological need, the Westernized lifestyle promotes a chronic lack of sleep, causing a variety of serious health problems. People deprived of sufficient sleep commonly suffer from mental illnesses, including panic attacks, anxiety, and depression. Sleeplessness causes the deterioration of brain functions as well as cardiovascular disorders [1], increasing the risk of developing diabetes and metabolic diseases. Sleep is a reversible biological process that is regulated by a dynamic interplay of neurotransmitters and neuromodulators in the brain. Surprisingly, one of the main sources of these metabolites resides in our gut and is far older than us: the gut microbiota.

Pioneering research suggests that an interplay between the gut microbial ecosystem and the sleep regulation pathways in the brain [1] is at the core of the association between sleep disorders and metabolic syndrome (MetS). Nevertheless, the precise biological mechanisms that govern this intricate network of neuroendocrine, immune, and metabolic pathways remain unknown. Beneath the surface of this captivating connection, many pages remain unturned, especially one of the main mediators in this struggle: the diet. Dietary patterns were linked to the inauguration of chronic disruptions of the circadian cycle and alterations rewiring the gut microbiome. Despite the importance of unraveling the impact of diet on metabolism and sleep, a global scientific overview has not been established in the literature yet. This review aims to provide a comprehensive overview, bridging the knowledge gaps of gut microbiota’s role in both MetS and sleep disorders and unveiling the common microbial associations between these seemingly unrelated chronic conditions.

## 2. Sleep Disorders

Sleep is a basic biological need. All living organisms have evolved an endogenous rhythm of sleep–wakefulness according to the light/dark cycle of approximately 24 h, known as the circadian rhythm [2]. This sleep/wake cycle is essential for the body to replenish and restore tissues, and to ensure proper functionality [3]. Based on physiological measurements, sleep is divided into two states with different functions: nonrapid eye movement (NREM) and rapid eye movement (REM) sleep. During NREM sleep, the body regenerates tissues and strengthens the immune system, while during REM sleep, dreams and memory consolidation occur. NREM sleep can be further subdivided into three stages. During NREM1, there is an individual transition from wakefulness to sleep. NREM2 is a phase of light sleep, which is followed by deep sleep (NREM3) and then the REM sleep phase [4].

The American Academy of Sleep Medicine (AASM) and the Sleep Research Society (SRS) recommend a regular sleeping period of seven or more hours per night for adults aged between 18 and 60 years. Correspondingly, the National Sleep Foundation (NSF) consensus report states that seven to nine hours are recommended for adults aged 18 to 64 years, while seven to eight hours are suggested for those 65 years of age or older [5].

Aiming to provide a consistent clinical and research assessment tool, Buysse and his team published the Pittsburgh Sleep Quality Index (PSQI) in 1989. The PSQI enables the self-assessment of sleep quality and disturbances over a period of one month [6]. The PSQI score evaluates the quality and quantity of sleep based on seven subjective scoring factors [6]. In addition to the PSQI score, clinicians and researchers refer to the third edition of the International Classification of Sleep Disorders (ICSD-3) for standardized clinical and research criteria to diagnose sleep disorders [6]. A comprehensive systematic review was recently conducted by Fabbri et al., validating the use of different self-reported questionnaires in assessing sleep quality. Despite the good internal reliability and validity of the PSQI score being reported, a clear definition and interpretation guidelines for the factor model underlying this tool should be recommended for clinicians [7].

Disorders in sleeping patterns are categorized based on clinical features [8]. Common sleeping disorders include sleep apnea, narcolepsy, insomnia, and circadian rhythm sleep disorders. Obstructive sleep apnea (OSA) is a sleep-related breathing disorder. Narcolepsy is characterized by daytime drowsiness and sudden sleep attacks. One of the most common sleeping disorders encountered in sleep medicine practices is insomnia. Insomniacs experience difficulties in initiating and maintaining sleep. The low sleep durations in insomniacs are caused by cognitive–emotional and physiological hyperarousal [9,10]. In a study conducted by Vgontzas and collaborators comparing young and old sleepers, age-related alterations in nocturnal wake time and daytime sleepiness were associated with elevations in both plasma IL-6 and cortisol concentrations in older sleepers [11]. Intriguingly, when focusing on insomniacs compared to age- and BMI-matched healthy adults, Vgontzas observed that the circadian rhythmicity of IL-6 and TNF-a secretion was disrupted, somehow explaining the daytime fatigue and performance decrements associated with insomnia [11]. Vgontzas et al. concluded that physiologic changes observed in insomniacs with short sleep durations are not a result of chronically accumulated sleep loss, but rather manifestations of an overall physiologic activation [11,12].

Sleep disorders linked to the circadian rhythm are often overlooked in sleep research. Circadian rhythm sleep disorders typically manifest as a misalignment between an individual’s biological sleep schedule and the 24 h physical/social and environmental cycle. People prone to developing circadian rhythm sleep disorders include people who work night shifts or have irregular work schedules and blind people [12,13]. These individuals experience an average of 4 h or less of sleep per 24 h period, which leads to excessive fatigue and a greater risk for workplace injuries and cognitive impairment [14].

The two most common sleep disorders that result from a disruption of the circadian rhythm are the advanced sleep phase (early onset, common in older adults) and the delayed sleep phase (later onset, common in teenagers). These two disorders are often misdiagnosed as insomnia or excessive sleepiness. However, circadian rhythm-related disorders are distinctly different disorders resulting from disturbances in sleep/wake cycle synchronization, while insomnia is a result of cognitive–emotional and physiological hyperarousal [14].

Multiple researchers have explored the linkage between circadian rhythms and mood disorders, including depression [15]. Indeed, typical symptoms of circadian rhythm disruption, like body temperature, plasma cortisol, norepinephrine, thyrotropin, and melatonin, are often observed in depressive patients [16]. The treatment of these disorders involves a pharmacological approach combined with light therapy and the establishment of a stable sleep/wake schedule [14]. Although rare, disturbance in the sleep–wake pattern can be found in people suffering from neurological disorders such as dementia, mental retardation, and brain damage, which often experience deep sleep both during the day and at night. The absence of distinct sleep patterns, along with difficulty in maintaining deep sleep, affect alertness/restlessness upon awakening and an inability to maintain a sufficient amount of sleep needed for their age [14].

As people age, sleep problems become more frequent [17]. On a biological level, sleep quality and patterns naturally change with age. In a meta-analysis, to develop normative sleep values across the human lifespan, Ohayon et al. covered the literature published between 1960 and 2003 and found quantitative sleep parameters to differ across age groups (children and adolescents between the ages of 5 and 19 years, adults aged 19–59 years, and elderly individuals aged >60 years) [18]. The total sleep time (TST) and sleep efficiency decreased with age. Additionally, the percentage of short-wave sleep (SWS, phase 3 deep sleep) and REM sleep was reduced in older participants compared to children and adults, while stage 2 sleep and sleep latency increased with age [19].

## 3. Exogenous and Endogenous Factors Influencing Sleep

Sleeping irregularities have become more common in today’s modern society. The modern day’s hectic rhythm often disrupts regular sleeping patterns. Factors associated with life in large cities, such as noctule light and noise exposure, shift work, fast food, medication, and social events, can influence the circadian rhythm negatively [19,20]. A vast cohort study of 224,986 Korean adults revealed poor sleep quality in 39.4% of participants, which positively correlated with unhealthy behavior such as smoking in both sexes under the age of 65 [21]. A similar study in Germany found sleep problems to occur in 36% of the general population [22].

Apart from negative lifestyle patterns such as smoking, alcohol consumption, and poor dietary habits, socioeconomic factors, including the living environment, were also attributed as major influential factors [19]. Educational and marital status, social isolation, and urban environments, including artificial light, air pollution, temperature, and noise, may affect sleep efficiency, quality, and latency and increase the risk of OSA [19].

A still not-well-known factor influencing sleep homeostasis is the gut microbiota, a complex microbial ecosystem that is established in the human intestine. The gut microbiome accounts for a mass of about ~10^13^–10^14^ microorganisms, including bacteria, fungi, protozoa, and viruses, overall harboring 50–100-fold more genes compared to its human counterpart. The mutual crosstalk between the metabolites produced during microbial fermentation and the host plays a central role in the regulation of human metabolic pathways. Despite the strong resilience of the microbial composition over a lifetime, lifestyle modifications, including unhealthy dietary habits, have been demonstrated to dysregulate the microbial diversity, a state called dysbiosis [23].

A vast number of diseases has been linked to the dysbiosis of the gut microbiota. The loss of gut microbial homeostasis not only contributes to the breakdown of peripheral intestinal functions, but also to central and enteric nervous system dysregulations. This bidirectional communication between gut microbial metabolism and brain functions is described as the gut–brain axis. Among several gut–brain affected pathways in disease status, there is the loss of sleep homeostasis, a main distinctive sign of multiple sleep disorders, including obstructive sleep apnea, parasomnias, narcolepsy, and restless leg syndrome [24]. In this complex scenario, lifestyle and dietary habits are important influencers in the maintenance of the internal biological clock, which is also intimately connected with the regulation of metabolic functions in the human body [23].

## 4. The Role of Host–Microbial Mechanisms in the Sleep Clock

An internal clock ensures the unfolding of a multitude of biological processes, starting from switching on and off genes up to the activation of a complex metabolic machinery inducing the release of hormones, digestive enzymes, and downstream metabolites in an elegant and systematized manner [25]. Multiple environmental factors such as meteorological changes, lifestyle, and dietary patterns influence this inner circadian clock. Intriguingly, not only do external signals give entrainment to this system, but it can align itself with the 24 h rhythm [26].

Human physiology alternates cycles of activity, characterized by higher energy expenditure, with recovery phases of sleep. Both of these phases rely on the internal synchronization of digestive processes and information processing by the brain. Therefore, dismembering this circadian pattern could lead to internal desynchrony and organ failure, as commonly observed in sleep disorders [27].

The body’s central circadian clock is located within the suprachiasmatic nucleus (SCN), which orchestrates the circadian rhythm throughout the entire organism [28]. The mammalian circadian clock is self-regulated using a feedback loop of a network of genes expressing proteins including CLOCK, BMAL1, Period circadian (PER1, PER2, PER3), and cryptochromes (CRY1 and CRY2) [29]. PER and CRY proteins interact in the presence of light exposure [30], forming a complex that relocates into the cell’s nucleus and binds to the transcription factor CLOCK-BMAL1, inhibiting sleep mechanisms at a cellular level in the waking hours. In contrast, during night time, the inhibitory complex of PER/CRY dissociates, resulting in a rhythmic transcriptional feedback loop regulating PER and CRY expression [30].

These circadian clock genes are directly coupled to genes of glucose and fatty acid metabolism, regulating processes influencing mitochondrial physiology. The circadian phosphorylation of dynamin-related protein 1 (DRP1) balances mitochondrial numbers through fusion and fission processes, thereby controlling the oxidative phosphorylation process and adenosine 5-triphosphate (ATP) production [31,32]. 

In addition to this central clock, peripheral organs can also affect sleep–wake regulation. The hypothalamic–pituitary–adrenal (HPA) axis is an endocrine pathway activated under stress conditions that plays a critical role in sleep regulation, as well as the bidirectional communication interconnecting the microbial crosstalk with the enteric nervous system (ENS) and the immune system in the gut with the central nervous system (CNS) via the vagus afferent nerve, overall defining the gut–brain axis [33]. 

## 5. The Neural System of the Gut–Brain Axis

The afferent vagus nerve can sense and recognize metabolic and immune activity through microbial neurotransmitters, hormones, fatty acids, and cytokines. Physiological and potentially harmful products in the gut can be transmitted to the brain through the intestinal and blood–brain barriers, providing another pathway of communication from the gut to the brain involved in the sleep–wake cycle [34].

Among the neuromodulators promoting the wakening cycle, there are acetylcholine (ACh), serotonin, dopamine (DA), norepinephrine (NE), hypocretin, and histamine, which produce excitatory effects on the tuberomammillary nucleus (TMN) [35]. In contrast, γ-aminobutyric acid (GABA), melatonin, adenosine, and glutamate are sleep-promoting amino acids, acting on receptors located in the ventrolateral preoptic nucleus (VLPO). Among these neuromodulators, 5-HT, ACh, norepinephrine, dopamine, melatonin, and GABA are also produced and metabolized by the gut microbes, which therefore directly and indirectly stimulate the interconnection of afferent neurons in the gut with the CNS. *Lactobacillus* spp. and *Bifidobacterium* spp. convert the excitatory neurotransmitter glutamate to the major inhibitory neurotransmitter GABA, which activates the GABAergic receptor system and modulates sleep disorders and memory [36,37], while *Clostridium sporogenes* contributes to the conversion of Trp to 5-hydroxy-tryptophan, which is then converted into the neurotransmitter serotonin, directly interacting with trace amine-associated receptors, enhancing its inhibitory neuroregulatory effect [38,39]. Also, a recent elegant research study demonstrated that the gut microbial metabolism mediates the neuroprotective effect of melatonin on sleep deprivation (SD)-induced cognitive impairment in mice. A potential biological mechanism has been proposed by Wang et al., suggesting the downregulation of *Lachnospiraceae NK4A136* and butyrate in the colon of SD mice and the inflammatory triggering role of both *Aeromonas* and LPS colonization [23]. The activation of the gut microbial metabolism by immune components can also be stimulated by this neural axis, as it happens with the biogenic amine histamine, secreted types of cells, and certain bacteria, including the *Moraxella morganii* and *Lactobacillus vaginalis* species. Histamine receptors are not only located in the CNS, regulating circadian and feeding rhythms, but also in peripheral organs orchestrating autoimmune reactivity [40].

## 6. The Role of the Immune System in the Gut–Brain Axis

Microbial-derived compounds constantly trigger an immune response in the gut, potentiating an inflammatory response, the activation of microglia from the enteric nervous system (ENS), and simultaneously interacting with vagal pathways [33].

The immune system in the gut unrolls fundamental tasks to guarantee gut homeostasis and a balance of the microbiota [41]. Microbes continuously stimulate immune cells, both directly, through microbial recognition via pattern-recognition receptors (PRRs) like TLR-like and NOD-like receptors, as well as indirectly, through the modulation of immune signaling through microbial products. For instance, beneficial *Lactobacillus rhamnosum* activates regulatory T-cells and suppresses the release of TNF-α from colon epithelial cells, which in turn prevents the expression of the pro-inflammatory cytokine IL-8l [42]. Not only do immune cells regulate the host–microbial crosstalk, but they also contribute to circadian clock regulation. Wang et al. demonstrated the ability of gut microbes to directly modulate the expression of important genes involved in circadian rhythm regulation, like *Rev-ERBA* and *Nfil3*, through the DC-ILC3-STAT3 immune pathway [43]. Similarly, the circadian expression of TLR9 by immune cells is also guaranteed through gut microbiota stimulation [44].

At the same time, microbial metabolites and bacterial cell wall components, like LPS, interact with the microglia cells from the innate immune compartment in the ENS, inducing an inflammatory cascade in the gut. In this regard, Zielinski et al. showed that LPS from Gram-negative bacteria can significantly reduce the power of the EEG theta, increasing the duration of NREM and decreasing the duration of REMS, thus inducing host fatigue [45].

## 7. The Role of Gut Microbiome in Metabolic Health

Dysbiosis of the gut microbiota accounts for the loss of microbial richness and diversity, giving rise to the prevalence of commensal, opportunistic pathogens over beneficial bacteria in the gut. The first findings concerning the role of gut microbiota in metabolic dysfunction were from Ridaura et al. In this study, an obesogenic role of the gut microbiota was demonstrated in mice [46]. Furthermore, a reduced microbial diversity was repeatedly observed in obesity and overweight, both in observational prospective studies and in randomized clinical trials [46]. Alterations in Firmicutes/Bacteroides and Prevotella/Bacteroides ratios were frequently observed in obesity, identifying the microbiota as a potential predictor for an increased body mass index (BMI) and fat mass [47]. Apart from a reduction in alpha diversity observed in obese individuals, a decrease in Christensenellaceae has been detected in obese compared to lean individuals as well as an inverse association with BMI [47]. 

Many key features of metabolic diseases, including obesity, type 2 diabetes, cardiovascular disease, and liver steatosis, propose a causative role of the gut microbial metabolism [48]. However, the mechanisms by which a specific microbial composition could exert its effects on the loss of energy balance experienced in metabolic disorders such as obesity are not fully understood, but several biological processes have been proposed. 

First is the contact-mediated mechanism of the innate immune system reacting against specific microbial molecular patterns, like TLRs and NOD-like receptors (NLRs), causing a local inflammatory response. Second, there is the indirect effect of microbial metabolism influencing both the intestinal epithelial integrity with the disruption of the tight junctions between epithelial cells, like zona occludens-1 (Zo1) and claudins, and the feedback regulation of energy metabolic pathways. Indeed, different metabolic diseases like obesity and diabetes are characterized by an increased gut permeability, known as a “leaky gut”, which co-exists with microbial dysbiosis. Despite the biological mechanisms behind this association still being unknown, a recent elegant study concluded that the reduced ability of an obese microbiota to metabolize ethanolamine instigates gut permeability inflammation by inducing miR-101a-3p, which in turn reduces Zo1. This reduced expression is in turn restored by re-establishing ethanolamine metabolism, surprisingly reverting glucose metabolic dysfunctions [49].

Thus, perturbations of intestinal homeostasis, along with increased intestinal permeability, are thought to make the way for microbial-derived compounds like endotoxins, lipopolysaccharides, and flagellins to enter the bloodstream, contributing to systemic inflammation.

Indeed, the loss of microbial richness and the predominance of pathogenic bacteria, commonly observed in metabolic disorders, are thought to take part in low-grade metabolic inflammation, establishing a source of sustenance for pathological processes such as insulin resistance and vascular dysfunction [50]. Indeed, the establishment of systemic inflammation, including high circulating levels of inflammatory cytokines (like TNF-a, IL-1B, 6, and 17), as well as an increased immune infiltrate in insulin-dependent tissues [50], without causing tissue damage, is the distinctive trait of metabolic disorders.

## 8. Biological Mechanisms Involved in Metabolic Syndrome

The term metabolic syndrome evolved from the very first findings ascribing a plethora of metabolic risk factors to atherosclerosis, up to the emphasis on insulin resistance as the main characteristic of MetS. Multiple clinical evidence has to be recognized to diagnose MetS, such as dyslipidemia, hypertension, and central obesity, apart from a disrupted insulin sensitivity. In this complex scenario, the presence of low-grade systemic inflammation is a well-recognized sign of microbial dysbiosis. Le Chatelier et al. first demonstrated that individuals with low gene richness in the microbiome are characterized by adiposity, insulin resistance, and dyslipidemia, and that gene counts correlate with metabolic parameters, like insulin levels and insulin resistance [51]. Another important confirmation was derived from the recent study from Asnicar et al. in the PREDICT1 cohort [52]), which confirmed the correlation of a specific microbiome composition with a large panel of cardiometabolic blood markers, including fasting and postprandial glycemic, lipemic, and inflammatory indices.

Overall, multiple studies agree that a reduction in butyrate producers, including *Faecalibacterium*, *Alistipes*, *Oscillobacter*, *Roseburia*, and *Pseudofavonifractor*, in MetS are key contributors to the pathogenic mechanisms of MetS [53]. Indeed, SCFAs, including butyrate, propionate, and acetate, have a particular affinity for G-protein coupled receptors, namely FFAR2 and FFAR3, which regulate satiety and intestinal motility by stimulating the secretion of glucagon-like peptide (GLP) and peptide YY (PYY) from intestinal L-cells [54]. Concurrently, activated G-protein receptors could also activate immune cells via the release of prostaglandin E2 and the expression of anti-inflammatory cytokine IL-10, as well as inhibiting deacetylation activity via NF-kB, thus regulating intestinal cell proliferation and differentiation.

However, contrasting results have been observed regarding higher circulating levels of SCFAs in metabolic diseases, like the study conducted by Perry et al., who demonstrated that a higher production of acetate by gut microbiota could activate the parasympathetic nervous system [55], causing an increased glucose-stimulated insulin secretion, ghrelin secretion, hyperphagia, and obesity [56]. Specifically, circulating butyrate levels correlated negatively with fasting plasma glucose levels, while propionate correlated positively with insulin sensitivity [57]. 

The pathophysiologic mechanisms underlying the development of MetS cover a wide variety of molecular dysfunctions, all contributing to the above-mentioned low-grade systemic inflammation, commonly observed in all metabolic disorders. Primarily, endothelial dysfunction initiates the atherosclerotic events that eventually lead to cardiovascular events and insulin resistance [50]. In MetS, the first signs of endothelial dysfunction have been observed in molecular alterations of hemostatic factors and fibrinolysis proteins, including plasminogen activator inhibitor-1 (PAI-1), low-density lipoprotein receptor-1 (LOX-1), and vasoconstrictor agent endothelin-1 (ET1), thereby causing vasoconstriction [58]. In parallel, adipose tissue inflammation with aberrant lipid accumulation [59], mitochondrial dysfunction with reactive oxygen species (ROS) generation and oxidative damage [60], and an autonomic dysfunction leading to the instauration of leptin resistance [61] all contribute to the cascade of metabolic impairments observed in MetS.

## 9. The Role of Gut Microbial Metabolism in Metabolic Syndrome

Beyond the explored contribution of the deficiency in SCFA producers in MetS, other microbial metabolites have been shown to play a critical role in the metabolic impairments observed in MetS. Among them, the decrease in bacterial deconjugation activity of primary bile acids has been frequently observed in MetS, with an increase in fecal levels of cholate and chenodeoxycholate, frequently associated with insulin and insulin resistance in MetS [62,63]. Secondary bile acids, apart from contributing to fatty acid absorption, are important signaling molecules in the liver, intestine, muscles, and brown adipose tissue through the activation of farnesoid X receptors (FXRs) and G-protein-coupled bile acid receptor 1, also known as TGR5. The signaling activated by FXRs regulates the expression of genes involved in the bile acid absorption and secretion in the gut.

Another important group of microbial-derived metabolites are branched-chain aromatic amino acids (BCAAs), including leucine, isoleucine, and valine, often associated with insulin resistance [64] and high-protein dietary patterns. The involvement of BCAAs in obesity-associated insulin resistance is thought to happen via an mTOR-dependent mechanism [65].

Other examples of detrimental microbial metabolites in MetS include trimethylamine *N*-oxide (TMAO) and indoxyl sulfate. TMAO, a microbial derivative of trimethylamine (TMA), has been clearly associated with increased cardiovascular risk factors, contributing to platelet hyperreactivity and enhanced thrombus potential [66]. Increased plasma levels of TMAO also correlate with enriched gut abundances of *Allobaculum* in a study conducted on mice [66]. At the same time, indoxyl sulfate is produced from tryptophan via gut microbial tryptophanase activity, leading to indole formation and subsequent toxic accumulation in the liver.

## 10. Dietary Modulation of Gut Microbiota in Metabolic Syndrome

The diet is believed to influence gut microbiota composition up to at least 20% of its variation in humans [67]. The diet acts as an environmental stressor to gut microbiome ecology, thus affecting both health and disease, including metabolic disorders, and even neurobehavioral traits. However, due to the homeostasis to a stable-state microbial profile, the impact of short-to-medium-term dietary interventions has only a transient effect on gut microbial composition [68]. Hence, only long-term dietary interventions may permanently alter the gut microbial composition; nevertheless, a consensus about its duration of intervention to affect a permanent change in composition has not been reached yet. An increasing number of studies further highlight the importance of feeding time and rhythmicity in shaping gut microbiota communities. An increasing number of studies are pointing out the importance of feeding time and rhythmicity in shaping gut microbiota communities [69]. 

Multiple studies conducted on both mice and humans have demonstrated that an increase in ingested dietary fibers leads to a higher prevalence of bacterial SCFA producers in the gut, known to beneficially act on glucose homeostasis. Therefore, is not surprising that unhealthy dietary habits, including a low intake of dietary fibers, are also associated with a higher risk of cardiometabolic disorders.

The predominance of detrimental microbial metabolisms in MetS is directly correlated to certain dietary patterns, such as the Western diet, which favors certain microbial processes over others. The analysis of the deeply phenotyped 1098 individuals from the PREDICT1 cohort (Table 1 [52]) revealed that adherence to Westernized dietary patterns strictly influences microbial composition and function, with the Clostridium-enriched cluster strictly linked to increased levels of lipoproteins. In individuals supplemented with barley kernel bread, there was a prevalence of the Bacteroides 2 enterotype, and an enrichment in *Prevotella copri* and *Blastocystis* spp. abundances were indicators of a favorable postprandial glucose metabolism. In contrast to these positive effects observed by Asnicar et al. in the case of *P. copri*, Pedersen et al. found that *P. copri* correlated with the production of branched-chain amino acids (BCAAs) that has been linked to type 2 diabetes, and that colonization with *P. copri* in high-fat-fed diet (HFD) mice induced insulin resistance, aggravated glucose intolerance, and augmented circulating levels of BCAAs [70,71]. This discrepancy is currently unclear but may result from strain-specific effects and different interactions with the diet. 

Clinical research was encouraged to evaluate the effects of fiber-rich foods on human metabolic health after studies in germ-free, obese mice that received fecal transplants from healthy mice showed improvements in obesity [46]. The dietary supplementation of fibers like inulin (Table 1 [72]) in pre-diabetic subjects, arabinose-xylooligosaccharides (AXOSs) in overweight subjects, or enriched-fiber foods like whole-grain and barley bread in healthy people, was enough to restore cholesterol levels and microbial dysbiosis in patients with metabolic syndrome (Table 1 [73,74,75,76,77]). Interestingly, the direct effect of fibers in ameliorating metabolic parameters in metabolic syndrome correlates with the enrichment of *Bifidobacterium* observed in the case of sleep improvements. MedDiet is characterized by the presence of a great source of dietary fiber, derived from a higher intake of cereals and vegetables. Also, the predominant intake of vegetable fats over fats of animal origin is a relevant characteristic of this dietary pattern, which is a major feeding source for the microbial counterpart. The role of MedDiet in MetS has been widely studied in different human cohorts. Particularly, in two independent cohorts, a specific enrichment of genera belonging to the Lachnospiraceae family was linked to improved cardiometabolic risk parameters (i.e., glucose and insulin resistance) (Table 1 [78,79]). Intriguingly, Galiè et al. [62,63] demonstrated that supplementation with a unique food source of fiber, like nuts, in the diet is not enough to observe significant improvements in cardiometabolic parameters, which comes with the mediation of specific bacterial genera, despite no great changes being observed in overall microbial diversity. The effect of nuts in MetS and gut microbiota composition has also been highlighted by Wang et al. (Table 1 [80]) in obese subjects, where no substantial changes in neither microbial richness nor body weight and waist circumference were observed. 

The potential beneficial effects of probiotics or symbiotics have also been tested in MetS and are reported here from three intervention studies [81,82,83]. A great role seems to be ascribed to the symbiotic treatments, with a beneficial effect on insulin concentrations and resistance, glucose levels, and BMI in MetS subjects [82]. The potential benefit of daily consumption of fermented foods like kefir was also tested in subjects with MetS, and no difference in metabolic parameters, including insulin, glucose, and cholesterol levels, was observed [84], nor did the gut microbiota composition experience great shifts in its composition.

**Table 1:** Associations between gut microbiota composition and metabolic syndrome in human clinical trials.

*N*	Authors	Title	Sample Size (*n*)	Disease Status	Type of Exposure	Methods	Outcome	Microbiota-Related Outcome(s)	Other Outcome(s)
1	Wang et al., 2021 [72]	Dietary Supplementation with Inulin Modulates the Gut Microbiota and Improves Insulin Sensitivity in Prediabetes	49	Pre-diabetes	Inulin	16S sequencing	Metabolic Syndrome/Gut Microbiota	*Bifidobacteriales*, *Bifidobacteriaceae*, *Bifidobacterium*, *Lactobacillae*, and *Lactobacillus* increased after inulin supplementation and improved lipid levels. *Eubacterium rectale*, *Butyricimonas*, and *Odoribacter* positively correlated with insulin and triglyceride levels.	Improvement in lipid parameters.
2	Ismael et al., 2021 [85]	A Pilot Study on the Metabolic Impact of Mediterranean Diet in Type 2 Diabetes: Is Gut Microbiota the Key?	9	T2D	MedDiet	16S sequencing	Metabolic Syndrome/Gut Microbiota	Increase in bacterial richness negatively correlated with glucose and insulin levels.	
3	Guo et al., 2021 [86]	Intermittent Fasting Improves Cardiometabolic Risk Factors and Alters Gut Microbiota in Metabolic Syndrome Patients	39	Metabolic Syndrome	IF	16S sequencing	Metabolic Syndrome/Gut Microbiota	*Acidobacteria bacterium*, *Eubacterium* sp. 1_3, and *Roseburia faecis* associated with improvements in cardiometabolic markers.	Improvement in cardiometabolic risk factors.
4	Marungruang et al., 2018 [87]	Improvement in cardiometabolic risk markers following a multifunctional diet is associated with gut microbial taxa in healthy overweight and obese subjects	47	Overweight	MFD	16S sequencing	Metabolic Syndrome/Gut Microbiota	Increased abundance of *Prevotella copri* in the MFD group as compared to the control group. Treponema correlated positively with blood pressure. In contrast, *Faecalibacterium* showed a negative association with blood pressure, while Bilophila appeared to associate with a negative blood lipid profile.	Improved blood pressure and lipid profile.
5	Haro et al., 2017 [88]	Consumption of Two Healthy Dietary Patterns Restored Microbiota Dysbiosis in Obese Patients with Metabolic Dysfunction	106	Obese	Healthy Dietary patterns	16S sequencing	Metabolic Syndrome/Gut Microbiota	Restoration of the gut microbiome dysbiosis.	
6	Wastyk et al., 2023 [81]	Randomized controlled trial demonstrates response to a probiotic intervention for metabolic syndrome that may correspond to diet	39	Metabolic Syndrome	Probiotic	16S sequencing	Metabolic Syndrome/Gut Microbiota	Metabolic shifts associated with a distinct microbiome profile.	Improvements in triglycerides and DBP in a subset of participants.
7	Gilijamse et al., 2020 [83]	Treatment with *Anaerobutyricum soehngenii*: a pilot study of safety and dose-response effects on glucose metabolism in human subjects with metabolic syndrome	24	Metabolic Syndrome	*Anaerobutyricum soehngenii*	Shotgun metagenomics	Metabolic Syndrome/Gut Microbiota	Gut microbiota alteration and change in bile acid metabolism. Levels of endogenous *Anaerobutyricum* spp. were not significantly different in fecal baseline samples when comparing the three dose groups (Kruskal–Wallis, *p* = 0.10).	Improvement in insulin sensitivity.
8	Sangouni et al., 2023 [89]	Garlic supplementation improves intestinal transit time, lipid accumulation product and cardiometabolic indices in subjects with metabolic syndrome: A randomized controlled trial	90	Metabolic Syndrome	Garlic Powder	16S sequencing	Metabolic Syndrome/Gut Microbiota	Garlic powder improved intestinal transit time.	Improvements in cardiometabolic indexes.
9	Eriksen et al., 2020 [74]	Effects of whole-grain wheat, rye, and lignan supplementation on cardiometabolic risk factors in men with metabolic syndrome: a randomized crossover trial	40	Metabolic Syndrome	Whole-grain rich diet	16S sequencing	Metabolic Syndrome/Gut Microbiota	WG rye is associated with higher *Bifidobaterium* and lower *Clostridium*. The effect of WG diets appeared to differ according to baseline microbial enterotype. WG rye resulted in higher abundance of *Bifidobacterium* lower abundance of *Clostridium* genus compared with WG wheat (FC = 2.58, *p* < 0.001) and baseline (FC = 0.54, *p* = 0.02).	WG rye, alone or with SDG supplementation, compared with WG wheat did not affect glucose metabolism but caused transient LDL-cholesterol reduction.
10	Rabiei et al., 2018 [82]	The Effects of Synbiotic Supplementation on Body Mass Index, Metabolic and Inflammatory Biomarkers, and Appetite in Patients with Metabolic Syndrome: A Triple-Blind Randomized Controlled Trial	46	Metabolic Syndrome	Synbiotic	16S sequencing	Metabolic Syndrome		Symbiotic treatment improved the status of BMI, FBS, insulin resistance, HOMA-IR, GLP-1, and PYY in patients with metabolic syndrome.
11	Velikonja et al., 2019 [75]	Alterations in gut microbiota composition and metabolic parameters after dietary intervention with barley beta glucans in patients with high risk for metabolic syndrome development	43	Healthy	Barley Bread	16S sequencing	Metabolic Syndrome/Gut Microbiota	Decrease in microbial diversity and richness in the test group. The pre-intervention gut microbiota composition showed higher abundance of health associated *Bifidobacterium* spp. and *Akkermansia municiphila* within cholesterol-responsive group.	
12	Xiao et al., 2014 [90]	A gut microbiota-targeted dietary intervention for amelioration of chronic inflammation underlying metabolic syndrome	93	Obese	WTP diet	16S sequencing	Metabolic Syndrome/Gut Microbiota	Improved lipid profile correlated to decrease in opportunistic pathogens of Enterobacteriaceae and Desulfovibrionaceae.	Improvement in insulin sensitivity and lipid profile.
13	Kjølbæk et al., 2020 [73]	Arabinoxylan oligosaccharides and polyunsaturated fatty acid effects on gut microbiota and metabolic markers in overweight individuals with signs of metabolic syndrome: A randomized cross-over trial	27	Overweight	AXOS	16S sequencing	Metabolic Syndrome/Gut Microbiota	AXOS intake has a bifidogenic effect and also increases butyrate producers in the gut microbiota.	No modulation of lipid or glucose metabolic parameters related to metabolic syndrome.
14	Wang et al., 2021 [80]	Gut Microbiota Composition is Associated with Responses to Peanut Intervention in Multiple Parameters Among Adults with Metabolic Syndrome Risk	209	Obese	Peanuts	16S sequencing	Metabolic Syndrome/Gut Microbiota	Minor modification of gut microbiome, except *Bilophila*, *Coprococcus*, and *Dorea*, appeared to be decreased after peanut intervention.	Decreased body weight, waist circumference and fasting blood glucose.
15	Akamine et al., 2022 [76]	Fermented brown rice beverage distinctively modulates the gut microbiota in Okinawans with metabolic syndrome: A randomized controlled trial	40	Metabolic Syndrome	Brown rice	16S sequencing	Metabolic Syndrome/Gut Microbiota	Increase in the number of beneficial species belonging to the Clostridia class, associated with reduced inflammation and increased SCFA production. Interestingly, ingestion of BA in contrast to WA resulted in a unique elevation in the abundance of number of beneficial species belonging to the Clostridia class, associated with reduced inflammation, and increased short-chain fatty acid production: *Lactobacillales bacterium* DJF B280 (*p* = 0.005), Butyrate-producing bacterium A2 207 (*p* = 0.012), and *Firmicutes bacterium* DJF VP44 (*p* = 0.038).	Reduction in inflammation.
16	Tian et al., 2022 [53]	Overall Structural Alteration of Gut Microbiota and Relationships with Risk Factors in Patients with Metabolic Syndrome Treated with Inulin Alone and with Other Agents: An Open-Label Pilot Study	60	Metabolic Syndrome	Inulin	16S sequencing	Metabolic Syndrome/Gut Microbiota	Inulin alone or combined with metformin or TCM altered specific gut microbiota taxa, like a decrease in Bacteroides, but not the general microbial diversity.	
17	Munch et al., 2019 [77]	Whole grain-rich diet reduces body weight and systemic low-grade inflammation without inducing major changes of the gut microbiome: a randomised cross-over trial	60	Healthy	Refined Grain	16S sequencing	Metabolic Syndrome/Gut Microbiota	Compared with refined grain, whole grain did not significantly alter glucose homeostasis and did not induce major changes in the fecal microbiome.	Whole-grain diet did not alter insulin sensitivity and gut microbiome but reduced body weight and systemic low-grade inflammation.
18	Hibberd et al., 2018 [91]	Probiotic or synbiotic alters the gut microbiota and metabolism in a randomised controlled trial of weight management in overweight adults	134	Overweight	Synbiotic (B420+LU) or Prebiotic (LU)	16S sequencing	Metabolic Syndrome/Gut Microbiota	Christensenellaceae was consistently increased in the LU and LU+B420 groups across the intervention time points.	LU+B420 correlated negatively to waist/hip ratio and energy intake at baseline, and waist-area body fat mass.
19	Bellikci-Koyu et al., 2019 [84]	Effects of Regular Kefir Consumption on Gut Microbiota in Patients with Metabolic Syndrome: A Parallel-Group, Randomized, Controlled Study	22	Metabolic Syndrome	Kefir	16S sequencing	Metabolic Syndrome/Gut Microbiota	Only Actinobacteria was significantly increased after kefir intervention.	Fasting insulin, HOMA-IR, TNF-α, IFN-γ, and systolic and diastolic blood pressure decreased after kefir intervention, not differently as with the control.
20	Thomas et al., 2022 [92]	Comparison between Egg Intake versus Choline Supplementation on Gut Microbiota and Plasma Carotenoids in Subjects with Metabolic Syndrome	23	Metabolic Syndrome	Egg Consumption	16S sequencing	Metabolic Syndrome/Gut Microbiota	Diet intervention had no effects on microbiota diversity measures or relative taxa abundances.	Bacterial biodiversity correlated with HDL.
21	Guevara-Cruz et al., 2019 [93]	Improvement of Lipoprotein Profile and Metabolic Endotoxemia by a Lifestyle Intervention That Modifies the Gut Microbiota in Subjects With Metabolic Syndrome	21	Metabolic Syndrome	Lifestyle Intervention	16S sequencing	Metabolic Syndrome/Gut Microbiota	A decrease in the dysbiosis of the gut microbiota associated with a reduction in the Prevotella/Bacteroides ratio and an increase in the abundance of *Akkermansia muciniphila* and *Faecalibacterium prausnitzii* after intervention.	Twenty-four percent reduction in serum triglycerides and a 44.8% reduction in MetS after a 75-day lifestyle intervention.
22	Galié et al., 2021 [62]	Effects of Mediterranean Diet on plasma metabolites and their relationship with insulin resistance and gut microbiota composition in a crossover randomized clinical trial	44	Metabolic Syndrome	Mediterranean Diet and Nuts	16S sequencing	Metabolic Syndrome/Gut Microbiota	Different microbial clusters that correlate with plasma metabolomics modules associated with improvements in cardiometabolic risk factors.	
23	Galié et al., 2021 [78]	Composition and Fecal Metabolites and their Relationship with Cardiometabolic Risk Factors	50	Metabolic Syndrome	MedDiet Nuts	16S sequencing	Metabolic Syndrome/Gut Microbiota	MedDiet induced higher abundances of *Lachnospiraceae NK4A136*, which correlated with insulin homeostasis.	Improvement in insulin resistance and glucose levels after MedDiet versus nut supplementation.
24	Tagliamonte et al., 2021 [61]	Mediterranean diet consumption affects the endocannabinoid system in overweight and obese subjects: possible links with gut microbiome, insulin resistance and inflammation.	82	Obese/Overweight	MedDiet	16S sequencing	Metabolic Syndrome/Gut Microbiota	Increase in *A. muciniphila* abundance in the gut independently of body weight changes.	Endocannabinoid tone and microbiome functionality at baseline drives an individualized response to an MedDiet in ameliorating insulin sensitivity and inflammation. The MedDiet intervention lowered plasma AEA (*p* = 0.02) and increased plasma OEA/PEA (*p* = 0.009) and OEA/AEA (*p* = 0.006).
25	Asnicar et al., 2021 [52]	Microbiome connections with host metabolism and habitual diet from 1098 deeply phenotyped individuals.	1098	Metabolic Syndrome	Observational	Shotgun metagenomics	Metabolic Syndrome/Gut Microbiota	An unfavorable microbial cluster, mainly including Clostridium species (*Clostridium innocuum*, *Clostridium symbiosum*, *Clostridium spiroforme*, *Clostridium leptum*, *Clostridium saccharolyticum*), positively correlated with lipoproteins associated with an increased risk of CVD and T2D	

Abbreviations: AEA, arachinodonoylethanolamide; AXOS: arabinoxylan oligosaccharides; BA: brown rice beverage; BMI: body mass index; CVD: cardiovascular disease; DBP: diastolic blood pressure; FBS: fasting blood glucose; FC: fold change; GLP-1: glucagon-like peptide 1; HDL: high-density lipoprotein; HOMA-IR: homeostasis model assessment-estimated insulin resistance; IF: intermittent fasting; LU: Litesse^®^ Ultra™ polydextrose (DuPont Nutrition and Health (Madison, WI, USA); MedDiet: Mediterranean diet; MFD: multifactoria diet; MetS: metabolic syndrome; OEA: oleoylethanolamide; PEA: palmitoylethanolamide; PYY: peptide YY; SCFAs: short-chain fatty acids; SDG: secoisolariciresinol diglucoside; T2D: type 2 diabetes; WG: whole grain; WTP diet: whole grains, traditional Chinese medicinal foods, and prebiotics diet.

## 11. The Dietary Modulation of Gut Microbiota in Sleep Disorders

The diet dependency of the chronobiology in gut microbiota was first tested by Leone et al. [94], who concluded that a high-fat diet disrupts the gene network of the circadian clock, as well as compromising the metabolic oscillation in microbe-produced metabolites (SCFAs and H_2_S). Particularly, the metabolic oscillation of butyrate was absent in HFD-fed mice as compared to low-fat-chow-fed mice, while the oscillation of a harmful metabolite like H_2_S was observed only in HFD-fed mice. The link between diet and sleep quality is reciprocal, as the circadian rhythm drives changes in metabolic homeostasis, and changes in the metabolic and nutritional status influence the circadian rhythm. Translating the findings observed in mice and observational studies in humans is a demanding need for establishing a causal relationship between sleep duration and GI disorders in which the gut microbiome could act as a mediator. In recent years, several controlled trials tried to address this need through an integrated approach of gastroenterologists and sleep specialists, as well as and the use of validated questionnaires and the ICSD-3 classification.

The dietary modulation of sleep homeostasis is regulated by gut microbial neuro-metabolites, which form a reservoir composed of metabolites and neurotransmitters affecting the sleep gut–brain axis. Protein-rich foods, for instance, contain compounds such as L-tryptophan (Trp) and alpha-lactalbumin (A-LAC), which affect the gut–brain axis [41]. Trp and A-LAC from protein-rich food or supplements are potential sleep-inducing agents [95,96]. To this respect, different studies have shown that the consumption of Trp-rich foods, such as milk, is linked to improved sleep quality [95,96].

In an attempt to unravel the role of dietary sources of Trp in stimulating the microbial metabolism involved in the GABA pathway, Schaafsma and collaborators performed a controlled, double-crossed, randomized clinical study with a dairy-based product supplementation as a good source of Trp. Three weeks of supplementation of the dietary product in subjects with disrupted sleep was effective to ameliorate their PSQI score and reduce their levels of cholesterol [56]. Intriguingly, these results seem to be driven by an increase in *Bifidobacteraceae* in feces collected at the end of the intervention period (Table 2 [56]). The contribution of intestinal *Bifidobacteriaceae* in the stress/anxiety/sleep axis is related to their ability to produce active GABA, for which a first step of bacterial proliferation is needed to obtain a greater increase in the microbial metabolism, which reasonably explains the longer time response following dietary supplementation. Similar findings on the role of *Bifidobacteriaceae* in promoting sleep homeostasis were obtained by Nishida et al. [97] with the probiotic supplementation of *Lactobacillus gasseri* CP2305 in young adults exposed to chronic stress (Table 2 [97]). An improvement in sleep quality was observed in these healthy patients coupled with an increase in *Bifidobacterium* spp. relative abundances and lower levels of *Streptococcus* spp., suggesting the cross-feeding role of this bacteria in inducing the beneficial *Bifidobacteriaceae* increase in sleep quality. Additionally, better sleep quality and duration were associated with fiber-degrading bacteria such as *Ruminococcus, Faecalibacterium* in two different observational studies conducted on a healthy population (Table 2 [98,99]).

## 12. Gut Microbiota: The Mediator between Metabolic Homeostasis and Sleep Quality?

In this review, we aimed to systematically report key findings associating metabolic syndrome and sleep disorder with gut microbiome modifications (Table 1 and Table 2). We performed a selective literature search on PubMed, reporting all the observational and randomized clinical trials evaluating the gut microbial composition in both MetS and sleep disorders in adult populations in the last 10 years. After a selective screening process based on both the quality and inherence of the studies to the review topic, we ended up with 11 studies relating gut microbiota with sleep disorders (Table 1) and 25 studies evaluating microbial signatures in MetS (Table 2).

Despite the individual variability in the analyzed cohorts, we observed a potential common microbial signature correlating with sleep disturbances in both healthy and unhealthy subjects, mainly pointing out the detrimental role of lower abundances of butyrate producers, especially Faecalibacterium, as well as an enrichment in the Bacteroidetes phylum. A similar pattern of the decrease in SCFA producers has been repeatedly observed in multiple cohorts of MetS, as summarized in Table 2. Since larger and more controlled cohorts of patients were available in the case of MetS, the microbial pattern identified is more consistent and taxonomically defined to be further investigated in the future. 

Fluctuations in the gut microbiota composition observed upon tailored dietary interventions may directly drive modifications in metabolic homeostasis, which in turn could both relieve sleep disturbances and ameliorate the insulin resistance pathway observed in MetS. A common feature observed both in MetS and sleep disruption is a decrease in butyrate producers, especially in *Faecalibacterium prausnitzii*, coupled with a reduction in some members of the Lachnospiraceae family, like *Roseburia*.

Intriguingly, dietary interventions based on healthy dietary patterns and fiber supplementation are sufficient to restore the enrichment of a butyrate producer like *Faecalibacterium prausnitzii*, as well as inducing a bifidogenic effect, which is linked with improved sleep quality and lipid levels [72]. Interestingly the higher abundances of specific members from the Lachnospiraceae family induced by the MedDiet seem to directly be involved in the known beneficial effects of MedDiet adherence on cardiometabolic health, especially on insulin and glucose levels [62]. 

Despite no studies, to the best of our knowledge, having directly evaluated the effect of the gut microbiome on sleep quality upon MedDiet adherence, several studies have evaluated the beneficial role of this dietary pattern in sleep disorders. Among them, a cross-sectional study on a large cohort of obese subjects from the PREDIMED-plus trial positively associated adequate sleep duration with beneficial effects on weight loss, BMI, and waist circumference with the MedDiet coupled with physical activity [106]. 

Similarly, Gianfredi et al. already demonstrated a significant correlation between sleep disorders and MedDiet adherence scores, coupled with BMI, in nursing students from the InSOMNIA trial [107]. Therefore, the main role of the MedDiet in ameliorating both sleep quality and metabolic parameters seems to have a common metabolic target: the insulin resistance mechanism. The role of insulin is probably ascribed to its augmented sympathetic activity in sleep restriction, which is related to a reduced utilization of brain glucose, elevated cortisol levels, and increased growth hormone secretion, with a consequent dysregulation of appetite control. Thus, determining the components of the gut microbiome that interact with this axis could be crucial for the design of tailored interventional approaches. Personalized nutritional interventions targeting this metabolic pathway could indeed add great value to the study of associated gut microbial changes.

In this regard, a new promising therapeutic approach is based on fecal microbiota transplantation (FMT), which recently emerged as a realistic alternative to probiotics for manipulating the gut microbiota in different chronic diseases. The safety and efficacy of FMT in the improvement of metabolic parameters in MetS and obesity have recently been systematically studied in a meta-analysis conducted by Proença et al. [108]. The findings from this meta-analysis showed that only two out of 12 studies achieved an improvement in metabolic parameters upon FMT. Thus, right now, there is not enough scientific evidence to support the use of FMT in clinical practice. Standardized procedures and more powered randomized clinical trials with longer follow-up times are therefore required to clarify the role of FMT and to investigate its potential useful role in the personalized treatment of sleep disorders.

In conclusion, we have highlighted the importance of following healthy dietary patterns with a higher content of dietary fiber to modulate the abundance of the major beneficial bacteria in the gut microbiota composition of subjects with MetS and/or sleep disorders.

This systematic review outlines a potential link between the gut microbiota and the common metabolic alterations found in metabolic syndrome and sleep disorders. However, further observational and mechanistic studies are required to confirm and validate these findings.

## 13. Methodology

**Figure 1:**
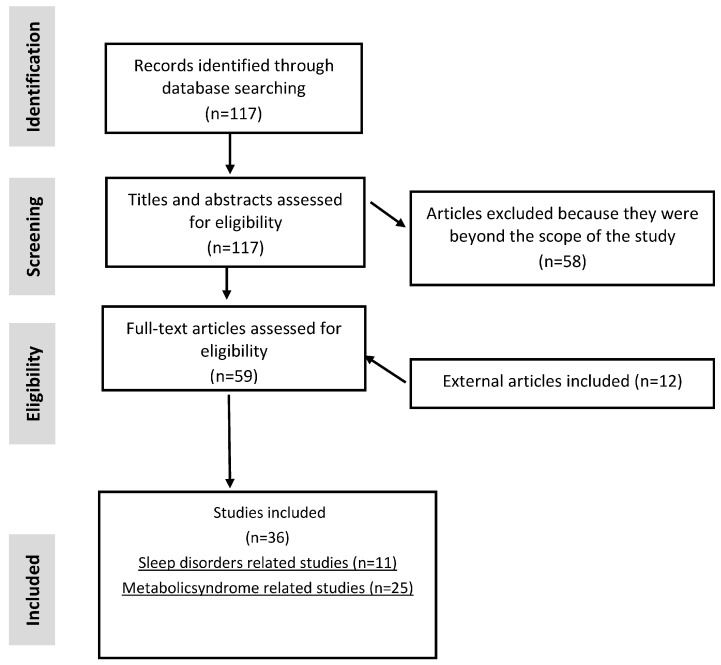
Study selection flowchart.

The selection procedure of the 36 articles discussed in Table 1 and Table 2 of the present narrative review was realized with two separate comprehensive literature searches in the Medline-Pubmed database, schematically represented in the flowchart in Figure 1. This study aimed to systematically review all the published observational studies and randomized clinical trials (RCTs) that investigated the microbial composition in adults with MetS and sleep disorders.

## 14. Eligibility Criteria

For the first search, publications that met the following inclusion criteria were selected:

- Dysbiosis and gut microbiome;
- Microbiome metabolites;
- Metabolic syndrome, sleep quality/efficiency, gut microbiome;
- Publication date range: 2013–August 2023;
- Language: English;
- Humans;
- RCTs and clinical trials.

## 15. Search Strategy

The search strategy for the articles illustrated in Table 1 and Table 2 was achieved using two main concepts: (1) gut microbiota AND metabolic syndrome or (2) gut microbiota AND sleep disorders. The overall process of literature selection is described in Figure 1. Respectively, 105 and 12 articles were selected at this phase for the first and second literature searches. Then, 117 articles were screened based on the presence of the eligibility criteria in the title and/or in the abstract. The present study and the corresponding search protocol is under registration in PROSPERO CRD42024497315 (http://www.crd.york.ac.uk/PROSPERO, accessed on 4 December 2023).

## 16. Data Extraction and Analysis

The texts of the 59 articles from the first screening procedure of the literature searches were extracted and carefully analyzed by two independent researchers. Moreover, the text of 12 other articles not selected in the literature search were found to be relevant for this review and were added to the second screening procedure.

The following exclusion criteria were considered for a second stringent screening:

- Non-original articles (reviews, meta-analyses, protocols and letters);
- Child populations;
- Cognition problems, mental illness, and unrelated disorders;
- Unclear outcomes;
- Gut microbiota composition with qPCR/RFLP;
- Mechanistic studies.

The most important information of each article (full-text) was extracted: authors, year, journal, population size, disease status, type of exposure, microbiota composition methods, microbiota outcomes, and other relevant clinical outcomes. Finally, 36 articles (25 for MetS and 11 for sleep disorders) were selected.

## 17. Study Quality Assessment

An external librarian was in charge of performing an unbiased first literature search and screening of the articles based on the inclusion criteria established above. A second and more stringent screening selection was performed by two independent researchers who evaluated the quality of each extracted text based on the guidelines provided by the PRISMA Statement [109]. A PRISMA checklist is provided in the Supplementary Materials.

## Figures and Tables

**Table 2:** Associations between gut microbiota composition and sleep disorders in human clinical trials.

*N*	Authors	Title	Sample Size (*n*)	Disease Status	Exposure	Methods	Outcome	Microbiota-Related Outcome(s)	Other Outcome(s)
1	Yang et al., 2021 [100]	Tianwang Buxin Granules Influence the Intestinal Flora in Perimenopausal Insomnia	13	Healthy	Tianwang Buxin Granules	Shotgun metagenomics	Insomnia/Gut Microbiota	Increase in *Roseburia faecis*, *Ruminococcus*, *Prevotella copri*, *Fusicatenibacter saccharivorans*, and *Blautia obeum* correlates with PSQI score.	Improvement in PSQI score: *p* < 0.05.
2	Zhanfeng et al., 2022 [101]	Regulation of sleep disorders in patients with traumatic brain injury by intestinal flora based on the background of brain-gut axis	28	TBI	Observational	16S sequencing	Gut Microbiota	Increase in Lachnospiraceae, *Bilophila*, *Odoribacter*, Bacteroidales, Bacteroidia, and Bacteroidetes (*p* < 0.05).	
3	Zhang et al., 2017 [102]	Human and rat gut microbiome composition is maintained following sleep restriction	11	Healthy	Sleep Restriction	16S sequencing	Gut Microbiota	No changes	
4	Gao et al., 2023 [98]	Sleep deprivation and sleep restriction resulted in downregulation of Faecalibacterium and butyrate abundance in the feces. Butyrate Ameliorates Insufficient Sleep-Induced Intestinal Mucosal Damage in Humans and Mice	22	Healthy	Sleep Restriction	16S sequencing	Sleep/Gut Microbiota	Downregulation of Faecalibacterium and butyrate producers, increase in Anaerostipes, Fusicatenibacter, and Veillonaceae associated with sleep restriction.	
5	Tang et al., 2022 [103]	Intermittent hypoxia is involved in gut microbial dysbiosis in type 2 diabetes mellitus and obstructive sleep apnea-hypopnea syndrome	27	T2DM+OSAHS	Observational	16S sequencing	Gut Microbiota	Decrease in Faecalibacterium, Eubacterium, and Lachnospiraceae.	The gut microbiota changes strongly correlated with the HCY, CRP, fasting plasma glucose, and hemoglobin A1c concentrations; AHI; mean oxygen saturation; and insulin resistance index in group T2DM + OSA (*p* < 0.05).
6	Grosicki et al., 2020 [99]	Self-reported sleep quality is associated with gut microbiome composition in young, healthy individuals: a pilot study	28	Healthy	Observational	16S sequencing	Sleep/Gut Microbiota	Increases in *Blautia* and *Ruminococcus* and lower abundances of Prevotella correlate with sleep quality.	
7	Yao et al., 2021 [3]	Relationships of sleep disturbance, intestinal microbiota, and postoperative pain in breast cancer patients: a prospective observational study	36	Breast Cancer	Observational	16S sequencing	Sleep/Gut Microbiota	At the phylum level, women with poor sleep quality had higher relative abundance of Firmicutes (*p* = 0.021) and lower relative abundance of Bacteroidetes (*p* = 0.013). At the genus level, women with poor sleep quality harbored a higher relative abundance of *Acidaminococcus* and a lower relative abundance of several genera.	
8	Schaafsma et al., 2021 [56]	The effect of a whey-protein and galacto-oligosaccharides based product on parameters of sleep quality, stress, and gut microbiota in apparently healthy adults with moderate sleep disturbances: A randomized controlled cross-over study	47	Healthy	Dairy-Based Product	16S sequencing	Sleep/Gut Microbiota	Relative abundance of Bifidobacterium increased (*p* = 0.02). Redundancy analysis showed an inverse relationship between baseline microbiota composition and baseline PSQI (*p* = 0.046).	Compared to placebo (skimmed milk), PSQI was only lower at day 14 in the 2nd intervention period in intention-to-treat (ITT) (*p* = 0.017; *n* = 69) and per-protocol (PP) (*p* = 0.038; *n* = 64) analyses. Post hoc analysis (modified PP: *n* = 47, with baseline PSQI ≥ 9, and endline day 14) showed a decrease in PSQI (−1.60 ± 2.53; *p* = 0.034).
9	Ho et al., 2021 [104]	Effects of lactobacillus plantarum ps128 on depressive symptoms and sleep quality in self-reported insomniacs: A randomized, double-blind, placebo-controlled pilot trial	40	Healthy	*Lactobacillus plantarum* (PS128)	16S sequencing	Sleep	Supplementation with *Lactobacillus plantarum* PS128 improved sleep quality (*p* < 0.05).	PS128 supplementation improved sleep quality.
10	Nishida et al., 2019 [97]	Health Benefits of Lactobacillus gasseri CP2305 Tablets in Young Adults Exposed to Chronic Stress: A Randomized, Double-Blind, Placebo-Controlled Study	60	Healthy	*Lactobacillus gasseri* (CP2305)	16S sequencing	Sleep/Gut Microbiota	Higher *Bifidobacterium* spp. and lower *Streptococcus* spp.	Intake of CP2305 improved sleep quality.
11	Zhu et al., 2023 [105]	Psychobiotic Lactobacillus plantarum JYLP-326 relieves anxiety, depression, and insomnia symptoms in test anxious college via modulating the gut microbiota and its metabolism	60	Healthy	Lactobacillus plantarum JYLP-326	16S sequencing	Sleep/Gut Microbiota	Higher Bacteroides and *Roseburia* and lower Prevotella and Bifidobacterium associated with sleep disturbances.	JYLP-326 improved sleep quality.

Abbreviations: AHI: highest apnea-hypopnea index; CRP: C-reactive protein; HCY: homocysteine; ITT: intention to treat treatment; OSA: obstructive sleep apnea; OSAHS: obstructive sleep/apnea hypopnea syndrome; PP: per-protocol; PSQI: Pittsburgh Sleep Quality Index; TBI: traumatic brain injury; T2DM: type 2 diabetes mellitus.

## Supplementary Materials

The following supporting information can be downloaded at: https://www.mdpi.com/article/10.3390/nu16030390/s1, PRISMA 2020 Checklist.
